# Supplementary material for: The worn-out syndrome: Uncertainties in late working life triggering retirement decisions
Source: PLoS One. 2023 Mar 29;18(3):e0282905. doi: 10.1371/journal.pone.0282905 (PMC10057830; doi:10.1371/journal.pone.0282905)
Supplement: S1 File — (DOCX) [file pone.0282905.s001.docx]

Interview guide for senior staff

**Introduction to the research project**

About the project:

- Research project at UCPH, Center for Health Research in the Humanities
- Investigating what working life is like in different workplaces.
- We interview senior employees, HR departments, managers, and colleagues.
- We use all the knowledge we gain from our fieldwork here to develop ideas on how to create better transitions from work to retirement with senior schemes. We're also publishing a podcast (radio programme) and a blog, and we're finalising the project by writing a report on what we've come up with.
- We're delighted that you want to help us with the research, so that we can hopefully create better retirement schemes that are just right for you, the people who need them.

About the interview:

- The interview will take a couple of hours and we'll talk about lots of different things, including how your working day goes, what makes you happy at work and what you want from a senior scheme.
- I'll guide you through all my questions. I'll ask questions along the way if there's anything I need clarification on. This is just to make sure I understand what you mean.
- Feel free to let me know if you need to take a break - we can just stop and start the interview again.
- I'll record the interview so I can concentrate on listening to what you say.
- You will be anonymous in our study, so only I and my closest colleagues on the research project will know that you have taken part. In the final report, you will not be recognisable.
- Do you have any questions before we get started?

**Theme 0: Introductory questions**

Would you like to start by telling us a little about yourself?

- Age, place of residence
- Family situation
- Employment status, title
- Educational background
- Work history
- Is there anything else important to know about you? (e.g. health, hobbies...)

How did you hear about our research project?

Theme 1: Work

Working day

- What does a normal working day look like for you?
- What are your work tasks?
- Which tasks do you like best?
- Which tasks do you like the least?

Job satisfaction (ask in general here. The theme will be elaborated later)

- What makes a working day good for you?
- What is the best thing about your job?
- What is the most important thing about a workplace for you?
  - Has this changed with age and experience?

Relationships at work (ask in general here. Theme will be explored later)

- What does it take to be a good colleague?
- What does it take to be a good boss?

**Theme 2: Seniority arrangements and senior schemes**

- When do you think you become a "senior" at work?
  - Are you a senior?
  - If not: When will you become one?
  - Do you have colleagues who are seniors?
  - Are you only senior at work?
- How do you feel about being one of the older employees?
  - Can you feel the difference? E.g. work assignments, community, collegiality, manager expectations, etc.?
  - Have you experienced discrimination because of your age?
    - At work or elsewhere?
- Do you know if there is a seniority scheme in your workplace?
  - What can you tell us about the seniority scheme?
- Do you know if anyone in the company has special arrangements because they are seniors?
  - How do these arrangements work?
- Have you talked to your manager about a senior scheme for you?
- If you could decide yourself, what would the senior scheme include?
  - For example, reduced hours, different tasks, flexibility, etc.?
- If you have a seniority scheme, how did it come about?
  - How long before you talked about a scheme had you been thinking about it?
  - Who initiated the conversation? (yourself, your manager, others)
- Does the nature of the MUS conversation change as you approach retirement age?

**Theme 3: Informant's own mental health**

- Are you looking forward to going to work?
  - What would it take for you to look forward to a day at work?
  - What would it take for you not to look forward to going to work?
- How would you rate your own happiness at work?
- When do you feel most useful at work?
  - When was the last time you felt useful at work?
- When do you feel comfortable at work?
  - When did you last feel comfortable?
  - And conversely, when did you last feel uncomfortable or even stressed?
- When do you feel competent at work?
  - How do you get to use your experience and skills as you get older?
- Is there anything now you are better at than 20 years ago?
  - And conversely, something you were good at 20 years ago that you are not good at anymore?
- Can you give an example of when you learned something new or did something new at work?
  - What about in your life other than work?
- Have you considered taking continuing education?
  - Have you talked to your manager about it?
- How do you feel about learning new things?
  - Has this changed with age?
- How do you feel about new colleagues?
  - How do you welcome them?
  - Is it a good way you do things?
- Do you feel able to form your own opinions at work, for example about tasks you are given or new initiatives?
  - How are you listened to in this respect?
  - Has this changed with age?
- How would you rate your own mental health?
- Who can do something to make you feel good or keep you feeling good at work?
  - What can you do yourself?
  - What can your manager do?
  - HR?
  - Trade union?
  - Works council/work environment representative?
- How has your job satisfaction developed with age?
- What do you immediately think of when I say "mental health"?

**Exercise 1: Draw your everyday life**

- Draw a day from when you get up to when you go to bed, explaining when you feel good and when you feel less good. Mark the times when you find it hard to cope with your tasks - or when it's less fun.

**Theme 4: Social well-being at work**

- How are your relationships with your colleagues?
  - How much do you talk to your colleagues during the day?
- When do you talk about work-related things and when do you talk about more social/personal things?
  - Can you try to describe a normal day in terms of colleague talk?
- Do you see your colleagues outside of work?
  - Why is this important/not important?
- How is your relationship with your manager?
  - Do you feel you can go to your manager if you have problems?
  - When was the last time you talked to your manager about a challenge?
- What makes a workplace good when it comes to social issues?
- Is it important to have a variety of ages in the workplace?
  - Why is this important/not important?
- Do you sometimes feel left out because of your age?
- Have you ever felt discriminated against because of your age?

**Exercise 2: The good working life as a senior**

*Introduce the exercise by saying that we are going to play a game and that the informant might know this from his/her grandchildren (be careful here and use the knowledge you have gained during the interview).*

The exercise is for the informant to write with a marker on a lego-block different elements that are important in the working life of a senior. For example, it could be "good colleagues" or "flexibility". The size of the block determines how important it is for the informant - if flexibility is very important, they should choose a large block. In the end, you have a number of blocks that together make up what it takes to have a good working life as a senior.

**Theme 5: Expectations for the future**

- What do you think about the future (e.g. in terms of work, family and interests)?
- When do you think, you will stop working?
  - Why do you think you will stop working?
    - Desire, physics, priorities, finances, etc.?
- What would be the worst way to stop working?
- What would be the best transition to retirement for you?
- Do you have examples of a good retirement transition?
  - E.g. with your friends, family, former colleagues, etc.?
- What do you think will happen at your workplace when you retire?
  - Are there knowledge or skills the workplace will miss?
  - Will you be missed?
- What do you think will be the most important thing for you to do when you retire?
- Do you talk to anyone outside of work about retirement?
- Have you talked to your union about retirement?
  - Why/why not?
  - What do you think they can help you with?
- Have you spoken to your pension fund about your pension?
  - Why/why not?
  - What do you think they can help you with?
- What do you dream about in relation to retirement? What do you fear?
- What will happen on your last day of work?

**Closing questions**

- Do you have anything else you would like to say?
- What is the most important thing you said during the interview/the most important thing I will take away?
- Is there anything you think I forgot to ask you about?
