# Supplementary material for: The worn-out syndrome: Uncertainties in late working life triggering retirement decisions
Source: PLoS One. 2023 Mar 29;18(3):e0282905. doi: 10.1371/journal.pone.0282905 (PMC10057830; doi:10.1371/journal.pone.0282905)
Supplement: Attachment — Submitted filename: PLOS ONE 2022-08-23 Worn-Out Syndrome.docx [file pone.0282905.s002.docx]

This is a clearly written argument about premature retirements and stereotypes of aging driving competent elderly employees out of the Danish labor force. The qualitative methods were conducted with appropriate rigor and the selection of quotations are well done and persuasive and moving. Indeed I was moved by the empathy of the researchers and consider the article to advance the field of aging studies and labor studies.

They note that they conducted extensive fieldwork in both production and finance sectors but they don’t present much or any of the production data. There mentions of the difference between production and finance are intriguing and are definitely worth a separate article. They might mention that to set up a future publication and also to explain why they bother telling us about the production findings which they do not fully develop but that are intuitive.

As an aging academic I resonated with the fear of perception of having weakening brain power that they discuss for the finance workers.

I would find it more interesting if in addition to the psychological/organization sociology contribution it explored a bit of the political economy logics and demographic shifts in social democratic and European countries that may be either pushing elder workers out or in of the labor force consistent with the psychological and sociological dynamics they identify in their interviews or possibly contradicting them? In other words are the elderly useful/needed from a profit-making productivity enhancing perspective or are they a counter productive dead weight to technology change and innovation. Also a bit more on whether the social democratic welfare/ retirement policies of Denmark specifically affect emotions and decisions about retiring from countries with a more savage form of capitalism such as the United States with less retirement benefits and fewer no limits on retirement ages and more elderly poverty.

I recommend publishing the article with minor revisions to reduce redundancy and my comments on political economy are optional—but I think they would make the paper much more interesting

to a wider audience if it did address political economy logics of somewhat objective productivity.

With respect to redundancy, the basic argument is repeated clearly in at least these four locations (see lines listed below). While it’s necessary to have some repetition of an argument in the abstract and in the conclusion and some of the discussion it gets a little bit boring reading it over and over. That said on a positive note their argument about the worn-out syndrome is very clear and persuasive and richly documented ethnographically and rings true to my personal experience as someone approaching retirement with trepidation:

- Abstract
- 72-75
- 518-521
- 595-603
