# Supplementary material for: The worn-out syndrome: Uncertainties in late working life triggering retirement decisions
Source: PLoS One. 2023 Mar 29;18(3):e0282905. doi: 10.1371/journal.pone.0282905 (PMC10057830; doi:10.1371/journal.pone.0282905)
Supplement: Attachment — Submitted filename: Response to reviewers.docx [file pone.0282905.s003.docx]

Rebuttal letter to the article Worn out syndrome

**Reviewer 1**

Thanks for your constructive comments, which points to some weaknesses in the manuscript that has helped us in improving it.

*- we have no numbers for how many managers and HR personnel were interviewed, I think the total of 92 covers them and employees and both sectors, although they are reporting mainly on the finance companies;* *- how the interviews were conducted and by whom, it would be helpful to have the semi-structured guide in an appendix;*

You are right that 92 covers them, but this was not obvious in the previous version. We have inserted a paragraph on lines 241-255 which describes how many of the different kinds of interlocutors we have followed, as well as how the interviews were conducted and by whom. We have included the interview guide in an appendix.

On lines 67-69 we describe why we are reporting mainly on the finance industry, and on lines 71-78 we now explain the Danish finance industry more in depth.

*- how were the interviews analysed, using a software programme? Were they coded? What was the coding process?*

You are right that this lacked in the previous version. We have inserted a new paragraph on lines 269-276 explaining our analytic approach and process.

*- what was the basis for selecting the direct quotations reported in the piece- were they indicative of widely expressed views or? The ethnographic vignette for example, why this case?*

In the new paragraph on lines 269-276 we explain that the selected quotes are indicative of widely expressed views unless noted otherwise.

On line 286-288 we now explain why we have chosen this vignette.

*The theoretical foundations for the piece and the literature review also need some attention. The article refers to meta stereotypes but it is not clear if the study was systematically looking for these or other manifestations of ageism in the interview process.*

In the new paragraph on lines 269-276 we explain that we use an analytic inductive approach. As such, the concept of meta stereotypes and the development of worn out syndrome were results of this analytic process and not something we knew we would explore from the beginning of the fieldwork. These concepts and phenomena appeared in our data and were subsequentially confirmed in the data.

*It would also have been useful to give a bit more attention to the literature on embodied/internalised ageism as this is what the data seems to reveal.
The notion of ‘worn out syndrome’ sounds very similar to the concept of ‘the decline narrative’ and it would have been useful to have some discussion of this. Also a number of authors have pointed to ‘too old for’ narratives which also sound similar. We need to know if worn out syndrome is different and if so how?*

Thanks for your suggestions. We had included some discussion on ageism in the discussion and literature review sections, but acknowledge that we could have shown how the concept relates to more literature than already included. We have therefore added on lines 192-195 and included some of the suggested literature – in particular the work of Vickerstaff and van der Horst as well as the ‘too old for’ narrative. However, we find that some of the suggested literature did not add to the argument.

*The article is tantalising in telling us about the two sectors but only really reporting on one. The difference between physical and cognitive ageing and how this is embodied would be especially interesting.*

We have mentioned the production industry for transparency purposes and in order to show how there is a difference between the politization of bodily decline on the one hand and cognitive decline on the other, which we show in the discussion lines 646-657.This could be interesting to explore further in a different article.

**Reviewer 2**

Many thanks for your constructive suggestions.

1. We agree that we needed a more thorough description of the Danish finance industry and have done so on lines 71-78. While we agree that the senior working life of traders could be interesting to investigate, we have no data on this particular group.
2. Most of the cases we have included in the paper have not retired but are approaching retirement age. As we show on lines 245-250, some of the interlocutors were managers, shop stewards and HR personnel, and from these data we have gained a glimpse into how other age groups perceive the senior employees. While this could be interesting to investigate further, this requires a different approach and a new fieldwork.
3. We are sorry that you think we miss concrete instances. With this kind of data we cannot show causal relations between the worn out syndrome and concrete actions, but we believe that we show concrete instances throughout the paper, in which the interlocutors describe when they experience uncertainty and a feeling of being worn out. To show this more clearly, we have repeated some of these instances in the discussion lines 599-617.

**Reviewer 3**

Thanks for your review and your appreciative reading of the manuscript.

We appreciate your suggestions regarding the production industry and have inserted how this could be explored in another article on line 695.

We have mentioned the production industry for transparency purposes and in order to show how there is a difference between the politization of bodily decline on the one hand and cognitive decline on the other, which we show in the discussion lines 646-657.

We sympathize with the comment on pursuing an article with a focus on the political economy of senior working life, but think that this requires a different article and scope. However, we are unfolding this more in an upcoming article stemming from the project.

While we do think that the Nordic welfare states create a different approach to retiring or staying in the workforce than in the US or countries with resembling state constructions, we cannot say much about this from our data. In the abovementioned upcoming article, we explore the literature about this issue in more depth.

Regarding your comment on redundancy, we agree that we have repeated the argument too much. We have therefore removed it from the lines 518-521 (in the new version, this would have been on line 587).
