# Supplementary material for: The worn-out syndrome: Uncertainties in late working life triggering retirement decisions
Source: PLoS One. 2023 Mar 29;18(3):e0282905. doi: 10.1371/journal.pone.0282905 (PMC10057830; doi:10.1371/journal.pone.0282905)
Supplement: Attachment — Submitted filename: Rebuttal letter.docx [file pone.0282905.s004.docx]

Dear reviewers and editor

Many thanks for your feedback and suggestions. Below we respond to each of the points raised.

***Reviewer 1:***

***I think it is bad practice not to say how many interviews were done in each of the two sectors***

We agree and have corrected this on lines 229-230 and 239-243.

***and although there is now more about why the finance sector is focused on it leaves open a set of questions about the productive sector. It is asserted that uncertainty was greater for white collar than blue collar but we are not presented with any data to back this up. It is counterintuitive as we would expect the manual workers to have more physical health conditions as they age and to be more uncertain about their physical ability to carry on working. I think it would make more sense to only discuss the finance sector as no data is really provided on the productive sector so the reader cannot assess any comparison being made.***

We have gone through the manuscript, and only find the production industry mentioned in the beginning (where we briefly present the initial project and fieldwork) and in the last section of the discussion. In this latter part, we have now removed some sentences, where we used data from the production industry. We now only reflect briefly on why we find the worn-out syndrome more in the finance industry on lines 626-643.

***We do not have any information of the interviewees pension status, there is a question asked: Why do you think you will stop working?
And ‘finance’ is provided as a prompt and a question about pension provider, I don’t know enough about pension arrangements in Denmark, perhaps everyone, whatever sector and work history, has adequate pensions but in most other places you would expect quite a difference between white collar and blue collar workers and those in finance would be likely to have very good pensions. Whilst finance may not be a major factor in retirement decisions, it is likely to be a boundary factor at least – can I afford to retire -. The fact that as state pension age has risen participation rates have increased would seem to underlie the importance of finance.***

Thanks for this comment. We agree that in other countries, worn-out syndrome might not play a role in retirement decisions, and have included this on lines 161-165.

***In the vignette case we hear nothing about her financial situation. It may be that for this relatively privileged group of finance sector workers money isn’t much of an issue?***

The vignette case does not include the financial situation, as this did not factor in her retirement decision, and was not the reason for including the vignette.

***The framing of the piece conceptually still raises some questions. The claim of the article overall is that the authors have discovered a new framing of age in the workplace the ‘worn-out syndrome’ (WOS) but if you substitute either ‘decline narrative’ or ‘too old for’ (already existing concepts) for WOS the overall argument works just as well. To put this another way how does WOS differ from these existing ideas in the literature? Substituting WOS really only works if there is a clear argument as to its superiority over existing concepts; in fact in the discussion and conclusion there is lots of talk about decline and some of the respondents converse in these terms as well.***

We agree that worn-out syndrome relates to the concepts mentioned, but believe that it differs as well, as we now explain more clearly on lines 186-191. , The ‘decline narrative’ and ‘too old for’ rely on internalized or embodied ageism, but WOS differs in the way that the participants do not have to believe, that they are worn out themselves, but the syndrome works just as well when participants are in doubt whether their surroundings might think they are worn out. Moreover, the ‘decline narrative’ and ‘too old for’ are not standard concepts that everyone in the field relates to, and there is plenty of space to develop new, relatable concepts with other nuances.

***Reviewer #2:***

***The authors have reviewed the paper following the reviewers' suggestions. I take their point about the differences between the impostor syndrome and the worn out syndrome. The way the data is presented and analysed makes it difficult to tell whether this syndrome is more specific to the financial services industry compared with other service sectors or not. We know that the interviewees work in banks but there is little insight in what they do and whether the specific profile of their expertise contributes to this syndrome or not. The only more specific information we get is about an interviewee working in the pensions department of one bank and about a new IT system being introduced. But this could be public administration too, or any other service sector introducing a new IT system. I am saying this because we could have two different claims: (1) that this syndrome is particularly present in the financial services industry, or (2) that this syndrome is present in the services industry, with the financial sector being just an example. If (2), which I believe is the case based on how the data is presented here, then the authors should make this clear and argue why they selected this sector for their investigations. We get a brief sentence that it has gone through restructuring since the 2000s, but so have other sectors presumably*** *too. I think this can be addressed with one or two sentences that make clear that the syndrome the authors discuss is not specific too or more pronounced in the financial services industry (it could be, but the authors do not have data to show this)*

Many thanks for this suggestion. We believe that the syndrome is widespread in other service sectors, but we do not have data to show this. On lines 67-70 we now state this as well as explain how the changes in the finance industry has led to issues with mental health.
